# Reconfigurable Memristive Quasi-Lumped Dual-Band Bandpass Filters

**DOI:** 10.3390/mi16070777

**Published:** 2025-06-30

**Authors:** Dejan Miljanović, Milka Potrebić Ivaniš, Ivo Marković

**Affiliations:** 1 Faculty of Technical Sciences, European University, 76100 Brcko, Bosnia and Herzegovina; miljande@teol.net; 2 School of Electrical Engineering, University of Belgrade, Bulevar Kralja Aleksandra 73, P.O. Box 35-54, 11120 Belgrade, Serbia; ivomarkovic@lbl.gov

**Keywords:** bandpass filter, dual-band filter, memristive switch, multilayer technology, reconfigurable microwave filter

## Abstract

This paper presents a dual-band bandpass filter with passband switchability controlled by using memristors. The memristor is a good choice as a control element due to its characteristics, such as low-power consumption, no bias needed, good electrical characteristics, and no moving parts. The filter’s reconfigurability is achieved by using memristors to selectively connect filter elements to ground. For the filter realization, multilayer technology with quasi-lumped elements has been chosen because of filter size miniaturization. Circuit-level simulations were initially used for quick analysis, followed by 3D EM simulations to validate the expected functionality of the proposed design concept. The results confirm the feasibility of a very small dual-band bandpass filter with independently controllable passbands. The frequency response of each of the two passbands (3.5 GHz and 5.8 GHz) can be tuned with negligible impact on the other passband by controlling the states of the memristors. The filter footprint area is equal to 0.10 λ_g_ × 0.12 λ_g_, where λ_g_ is the guided wavelength at 3.5 GHz.

## 1. Introduction

As 2030 approaches, the time has come to accelerate the development of the sixth-generation (6G) network. Nowadays, intelligent communications are supported by 5G networks, enabling applications such as smart buildings, virtual reality (VR), augmented reality (AR), and the Internet of Things (IoT). These use cases require peak data rates of around 20 Gb/s, latency up to 1 ms, and operate in the Sub-6 GHz and mmWave frequency bands [1,2].

The 6G network must support applications such as Internet of Everything (IoE), tele-robotics, tele-driving, tele-education, and extended reality (XR, besides AR and VR). These applications require higher data rates of up to 1 Tb/s, latency below 1 ms, reliability 10 times greater than for 5G, reduced network energy consumption, integration of space, aerial, ground, and undersea communication networks, and the use of novel frequency bands such as THz and visible light spectrum [3,4]. The introduction of these higher frequency bands requires the development of novel technologies to implement front-end devices such as antennas, filters, amplifiers, oscillators, mixers, switches, etc. In order to design switchable and reconfigurable devices, such as filters and phased-array antennas, RF switches are used as a primary component to (dis)connect system parts or tune a specified frequency band. An RF switch is typically modeled as a capacitor and a resistor connected in parallel.

Conventional implementations of RF/microwave switches require a DC bias to preserve ON/OFF states along with dynamic energy during switching transients. Traditional RF switches include PIN diodes, MEMS, and FET implementations. These switches offer high switching speeds, high energy efficiency, and compatibility with CMOS technology.

Furthermore, non-volatile RF/microwave switches, such as RF memristors, do not require a DC bias for ON/OFF states, thereby increasing the energy efficiency of RF/microwave systems. Various switching technologies have been investigated, with the most promising implementations based on two-dimensional (2D) materials [4]. Non-volatile switch realizations offer high switching speeds and cutoff frequencies, minimal energy consumption, nanoscale layouts, and integration potential with CMOS technology.

In 2015, Pi et al. fabricated the first non-volatile RF nano-sized switches (memristive switches), demonstrating excellent RF characteristics: an insertion loss of 0.3 dB in the ON state, 30 dB isolation in the OFF states at 40 GHz, and an operational frequency reaching up to 110 GHz [5]. Subsequently, Ge et al. [6] introduced the concept of “atomristors,” realizing non-volatile resistive switching in monolayer transition metal dichalcogenides (TMDs) such as MoS_2_, WS_2_, MoSe_2_, and WSe_2_. They demonstrated the universality of memristive behavior in atomically thin sheets. Their devices operated at frequencies up to 50 GHz, provided 12 dB isolation in the OFF state, and introduced insertion loss of around 1 dB in the ON state. In [7], Hus et al. designed atomristors which achieved results that could satisfy 6G demands: operating frequencies of up to ~0.5 THz, and data rates of around 100 Gbit/s. One of the most recent studies [8] presents an ultra-fast atomically thin memristor based on 2D hexagonal boron nitride (h-BN), achieving switching times of 120 ps to 3 ns, with only ~2 pJ required per switching event and a CMOS-compatible frequency range. These findings highlight the strong potential of 2D RF switches in next-generation communication systems.

Over the past decade, researchers proposed many use cases of RF memristors, such as: amplifiers [9,10], antennas [11,12], attenuators [13], filters [2,14,15], oscillators [16], phase shifters [2], reconfigurable intelligent surfaces (RISs) [17,18], etc.

Relevant previous works of the authors are available in the literature. For example, a loaded-line phase shifter utilizing a memristor as a low-power, non-volatile switching element was presented in [2], enabling discrete phase control without continuous biasing. A memristor-based attenuator offering precise and programmable amplitude control was also demonstrated in [13], highlighting the potential for compact, reconfigurable gain adjustment. Additionally, a dual-band filter based on a multilayer folded structure was proposed in [14], where memristors were employed to dynamically switch between frequency bands.

This paper presents a novel reconfigurable dual-band bandpass filter using RF memristors. By leveraging advantageous properties of memristors and the compact nature of planar multilayer technology, the proposed design achieves significant miniaturization along with reconfigurability. A compact reconfigurable dual-band bandpass filter based on multilayer quasi-lumped resonators and RF memristive switches is designed and analyzed. The filter comprises two parallel resonator networks operating at 3.5 GHz and 5.8 GHz, targeting applications such as 5G and Wi-Fi. By selectively grounding resonators through memristors in the ON state, individual passbands can be suppressed or activated, enabling four operating modes: dual-bandpass, low-bandpass, high-bandpass, and bandstop. Memristors are modeled as low-value resistors in the ON state and as capacitors with low capacitance in the OFF state. The proposed design is first analyzed using an equivalent circuit model, and then verified through full-wave 3D electromagnetic (EM) simulations. In the final phase, the memristor-based filter is compared to PIN diode-based realization. The results confirm that the memristor-based approach offers good potential for low-power, reconfigurable RF front-end systems.

The remainder of this paper is organized as follows. In Section 2, a multilayer quasi-lumped resonator and its corresponding equivalent circuit model are briefly presented. Section 3 introduces a single-band bandpass filter based on multilayer quasi-lumped resonators, along with its equivalent circuit model, which is used to observe the impact of parameter variations on the filter’s characteristics. In Section 4, a reconfigurable dual-band bandpass filter design using memristors as switching elements is described and analyzed in detail. The use of PIN diodes instead of memristors is examined in Section 5. Concluding remarks are given in Section 6.

## 2. Resonator Structure

Microwave bandpass filters implemented with quasi-lumped elements, using the planar multilayer technology, have been thoroughly analyzed in the literature [19]. Detailed analyses of resonator structures, feeder implementations, and inter-resonator coupling types have demonstrated the viability of this approach for the realization of bandpass filters. The most important facts of this filter realization will be briefly presented.

**Figure 1:**
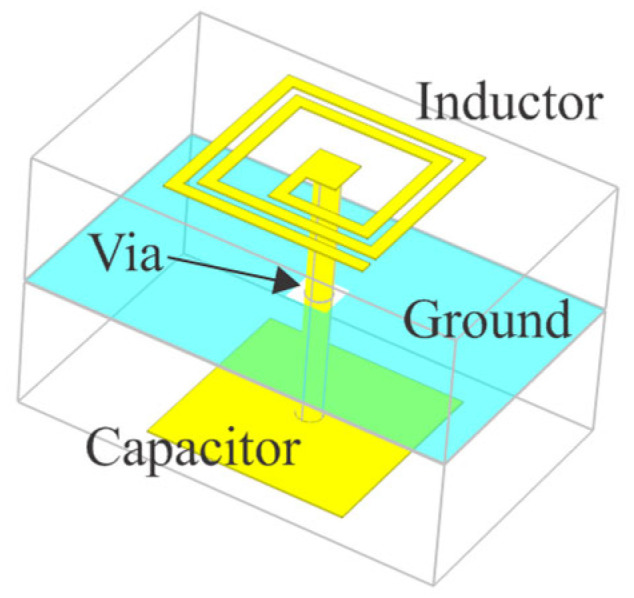
The 3D EM model of the multilayer quasi-lumped resonator.

The fundamental part of the filter is the resonator, which is realized as a multilayer quasi-lumped resonator implemented on a double-sided microstrip, as shown in Figure 1.

The resonator comprises three elements: a spiral inductor, a via, and a parallel-plate capacitor. The spiral inductor and capacitor are fabricated on opposite sides of conductive substrate layers, while a common ground plane is placed between the two substrates. The via connects the inner end of the spiral inductor on the top plane and the capacitor at the bottom plane without an electrical connection to the ground plane. An important characteristic of the adopted spiral inductor realization is that the outer end is open-ended. This is very important because it defines the way the whole structure behaves. The inductor has zero current density at the outer end, but the electric charge accumulation increases toward this open end. As a result, the inductor exhibits enhanced capacitive coupling to adjacent elements, compared to the case when both ends of the inductor are connected to other elements. This property is critical for enabling effective inductor-to-capacitor coupling in the design. Furthermore, the open-ended layout of the inductor is well-suited for implementing switchable resonators. The concept behind this reconfigurability will be shown in more detail.

**Figure 2:**
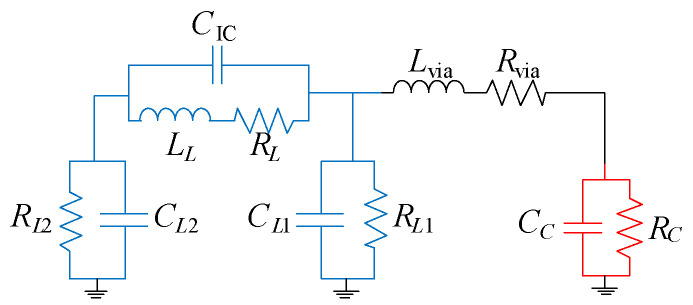
Equivalent circuit model of the multilayer quasi-lumped resonator.

The equivalent circuit model of the resonator is shown in Figure 2. An accurate equivalent circuit allows for quicker analysis and provides more intuitive insight into the resonator’s behavior compared to the full EM simulation.

The square-spiral inductor (marked in blue) is modeled using a π-type electric network with the following parameters: inductance (*L_L_*), resistance (*R_L_*), parasitic capacitances, and resistances to ground (*C_L_*_1_, *C_L_*_2_, *R_L_*_1_, and *R_L_*_2_), capacitance between the turns of the spiral inductor i.e., inter-spiral capacitance (*C*_IC_).

The cylindrical via is modeled with an inductance (*L*_via_) and resistance (*R*_via_) while the capacitor is modeled with capacitance (*C_C_*) and resistance (*R_C_*) to ground.

A simplified equation for the resonant frequency of the resonator (neglecting via inductance and resistive losses) can be written as [19]:

(1) $$f_{0}=\frac{1}{2\pi \sqrt{L_{L}\left(\frac{\left(C_{C}+C_{L1}\right)C_{L2}}{\left(C_{C}+C_{L1}\right)+C_{L2}}+C_{\mathrm{IC}}\right)}},$$

by examining the influence of each parameter in Equation (1) on the resonant frequency, one can identify which parameters can be modified to make the resonator switchable and suitable for use in a controllable filter. The same conclusions can be drawn more easily by simulating the equivalent circuit model of the multilayer quasi-lumped resonator. This simulation-based approach using an equivalent circuit model has been adopted and will be presented in the following sections.

## 3. Bandpass Filter and Electrical-Circuit Model

**Figure 3:**
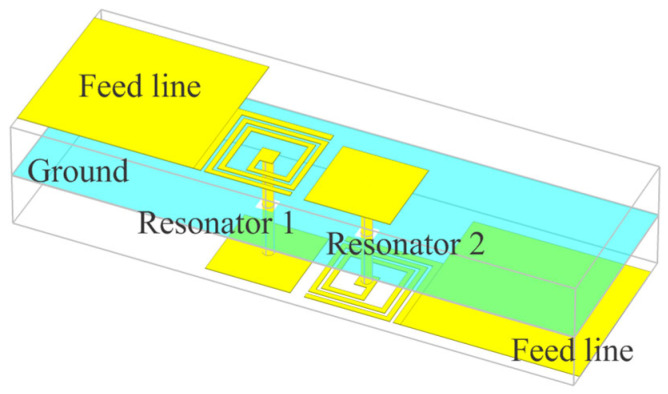
The 3D EM model of the second-order bandpass filter with inductor-to-capacitor coupling.

A single band bandpass filter can be designed using two of these miniaturized resonators, mutually coupled and appropriately fed. An example of this design approach is shown in Figure 3.

**Figure 4:**
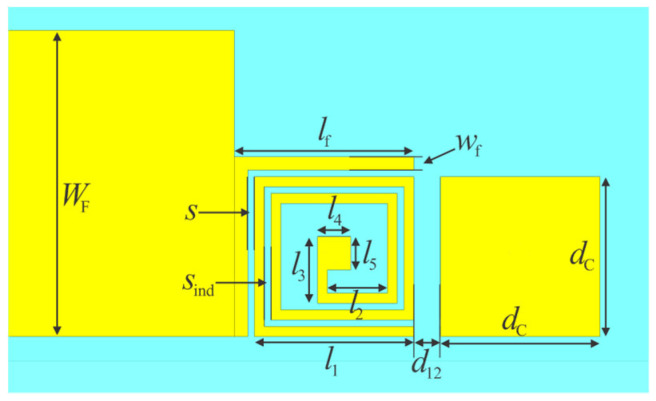
Structure of the second-order bandpass filter.

The bandpass filter was designed for a center frequency of 3.5 GHz. The physical parameters of the RT/duroid 5880 substrate used in the design are as follows: relative premitivity ɛ_r_ = 2.2, loss tangent tan δ = 0.001, thickness *h* = 1.575 mm, copper cladding *t* = 18 µm. The conductor conductivity was set to 20 MS/m to account for losses due to surface imperfection. The via-hole is realized as a cylindrical via with a diameter of *D*_via_ = 0.3 mm. The dimensions of the filter, as detailed in Figure 4, are listed in Table 1.

The dimensions presented in the table represent the final values obtained after tuning. The initial dimensions were determined for individual quasi-lumped elements. Due to mutual couplings between elements, these final dimensions were determined through 3D electromagnetic simulations of the complete filter structure.

The coupling between resonators was realized as inductor-to-capacitor coupling on both sides of the multilayer structure, as this type of inter-resonator coupling always generates transmission zeros. The existence of transmission zeros improves the filter’s selectivity.

**Figure 5:**
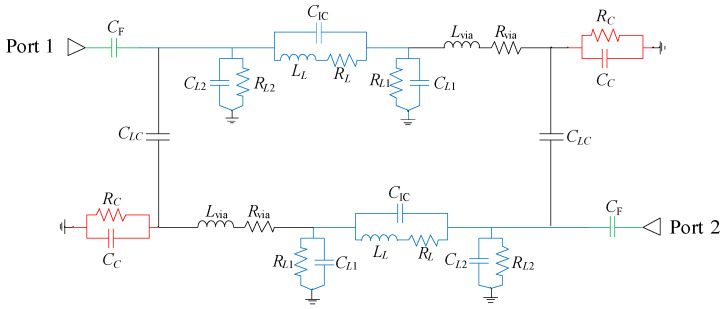
Equivalent circuit model of the second-order bandpass filter with inductor-to-capacitor coupling.

As previously stated, the open-ended inductor results in a predominantly capacitive coupling between the resonator elements (i.e., the inductor and capacitor). For the same reason, the coupling between the feeder and the inductor is also primarily capacitive, rather than inductive or conductive. Taking these factors into account, an equivalent circuit model of the second-order bandpass filter with inductor-to-capacitor coupling has been derived (Figure 5).

The values of elements used in the circuit model from Figure 5 are listed in Table 2. Initial estimates and value ranges were obtained using closed-form expressions for calculating the inductance and resistance of planar spiral inductors, the capacitance of patch capacitors, the inductance and resistance of vias, and the capacitance of coplanar coupled conductors. These values are determined by physical dimensions and layout of the elements (the number of turns, outer diameter, spacing between turns, turn width for the inductor; outer dimension for the patch capacitor; the spacing and length for coplanar coupled conductors). The inter-turn (intercoil) capacitance of the inductor was estimated through parameter extraction, based on the inductor’s 3D EM model. In the final step, small adjustments to the element values in the circuit model were made to match the results of the single initial 3D EM simulation. These refinements account for parasitic capacitances that are difficult to model analytically. As a result, the proposed circuit model depicts the influence of each parameter on the real filter characteristics very accurately, while significantly reducing computation time compared to full 3D EM simulations. This approach has been extensively used to evaluate the feasibility of implementing a reconfigurable bandpass filter.

**Figure 6:**
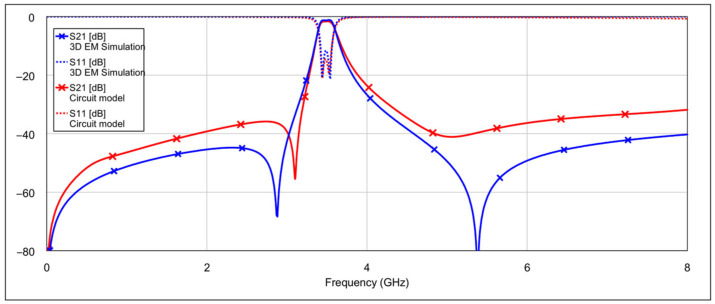
Frequency responses of the 3D EM simulation and circuit model of the proposed bandpass filter.

The frequency responses obtained from the 3D EM simulation and the circuit model of the proposed bandpass filter are shown in Figure 6.

The equation for the resonant frequency of the loaded resonator, based on the presented circuit model (see Figure 5 with *C_LC_* = 0, [19]), and neglecting via inductance and losses, can be expressed as:

(2) $$f_{0}=\frac{1}{2\pi }\sqrt{\frac{\left(C_{C}+C_{L1}+C_{L2}+C_{F}\right)}{L_{L}\left(\left(C_{C}+C_{L1}+C_{\mathrm{IC}}\right)\left(C_{L2}+C_{F}\right)+\left(C_{C}+C_{L1}\right)C_{\mathrm{IC}}\right)}}.$$

The resulting Equation (2) is relatively complex, making it difficult to draw straightforward conclusions about the relative influence of each parameter on the resonant frequency. The idea behind making the resonator switchable is to identify which parameter should be changed to eliminate the resonant frequency within the band of interest. This can be achieved either by eliminating the resonance effect entirely or by shifting it to higher frequencies that fall outside the relevant operating range.

For example, from Equation (2), it is obvious that making the value of the inductor inductance negligible (tending to zero) or very high (tending to infinity) would shift the resonant frequency towards infinity or zero value, respectively. In contrast, performing a similar analysis for other parameters is less straightforward and involves more complex relationships.

A much easier and faster approach is to use a circuit model within a circuit simulator to interactively observe the impact of parameter changes on the filter’s characteristics. This approach has been used as the first step to identify a set of parameters that could potentially be adjusted or modified. However, it is important to remain mindful of physical constraints and the practical feasibility of implementing such changes in the actual design.

The analysis showed that the resonant frequency is most significantly influenced by the inductance of the inductor, the inductor’s capacitance to the ground, and the capacitance of the capacitor. Since the memristor was chosen as a switching element, its effect was evaluated by placing it at strategic locations within the filter. The placement of the memristors was guided by several considerations: proximity to the element/parameter to be changed, minimizing occupied area, easy access to the ground layer, easy access for additional circuitry for memristor control, and overall design simplicity. Based on these criteria, we decided to place memristors as switches to the ground at two locations: the outer end of the inductor and at the capacitor.

The RF memristor is a nanoscale device which is not yet commercially available as a lumped component, unlike traditional elements: resistors, capacitors, inductors, and other semiconductor devices. Currently, only their characteristics and equivalent circuit models are available in the literature, and these are used for analysis and device design purposes. Once memristor technology becomes widely commercially available, it is expected that memristors will be offered in standard SMD (surface-mount device) packages. With this in mind, we have chosen to adopt the footprint area of an SMD package 0603 (0.6 mm × 0.3 mm) and corresponding pad dimensions in our design.

**Figure 7:**
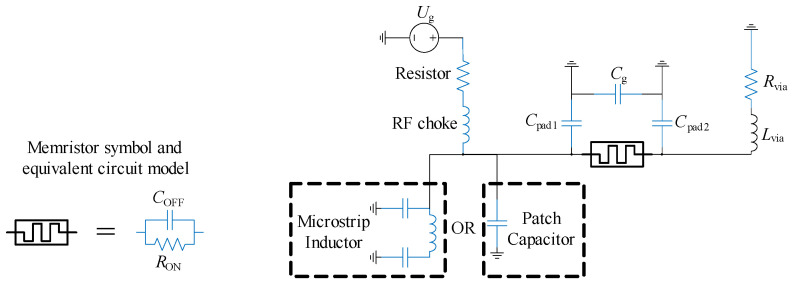
The memristor equivalent circuit model and its programming circuit.

The equivalent circuit model that accurately represents the memristor consists of a parallel connection of a capacitor and a resistor, as shown in Figure 7 [5]. This equivalent circuit model is proposed by analyzing the statistical analysis of more than 30 measured memristors (see [5]). *R*_ON_ represents the equivalent resistance of the memristor in the ON (conducting) state, while *C*_OFF_ represents its capacitance in the OFF (non-conducting) state. The average values of *R*_ON_ and *C*_OFF_ are 3.6 Ω and 1.37 fF, respectively. The memristor transitions from the two states when a voltage pulse of appropriate polarity is applied. For example, applying a voltage of 3 V or higher switches the memristor to the conducting state, while a voltage of –0.4 V or lower switches it to the non-conducting state [5]. These transitions can occur at any point during filter operation, with the added advantage of a very fast state change, less than a nanosecond. Unlike other commonly used switching elements, such as PIN diodes, the memristor does not require a continuously biased circuit for operation. The proposed control circuit for programming the memristor consists of a series connection of a resistor (10 KΩ), an inductor—RF choke (30 nH) and a voltage source (Figure 7). The roles of capacitors *C*_pad1_, *C*_pad2_, and *C*_g_ will be explained later. During programming, a current path is formed through the sequence: voltage generator—resistor—RF choke—memristor-via-ground plane. The resistance and inductance of the via are significantly smaller than those of the programming resistor and RF choke. Consequently, the memristor acts as a low-value resistor or low-capacitance capacitor between the printed inductor/patch capacitor and ground, as shown in Figure 7.

**Figure 8:**
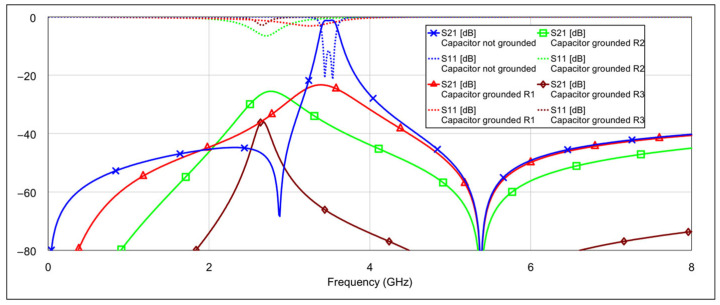
Influence of grounding the capacitor with various values of resistances *R*_1_ = 500 Ω, *R*_2_ = 100 Ω, *R*_3_ = 3 Ω (where *R*_3_ = 3 Ω corresponds to memristor ON state).

**Figure 9:**
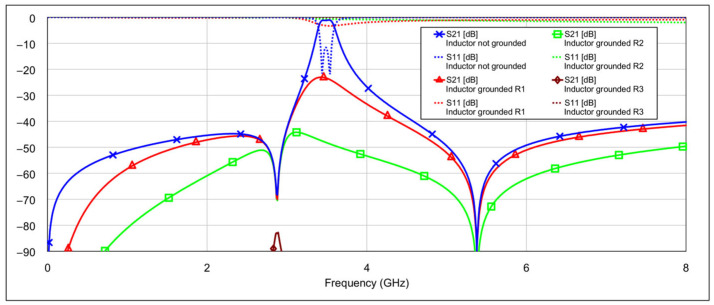
Influence of grounding the inductor with various values of resistances *R*_1_ = 500 Ω, *R*_2_ = 100 Ω, *R*_3_ = 3 Ω (where *R*_3_ = 3 Ω corresponds to memristor ON state).

The simulation results of the second-order bandpass filter with inductor-to-capacitor coupling, using the circuit model and varying the states of the memristors at the selected positions, are presented in Figure 8 and Figure 9.

The blue traces in both graphs represent the filter’s frequency response with all memristors in the OFF state. The other traces in Figure 8 correspond to the case where the patch capacitors of the resonators are grounded through different resistance values. The final brown trace corresponds to the resistance of 3 Ω, which is the memristor’s resistance in the ON state. The additional resistance values are included for comparison to illustrate how varying resistance levels affect the filter response. Similarly, the traces in Figure 9 show the case when the inductors of the resonators are grounded through different resistance values. The interpretation of these traces follows the same logic as in Figure 8.

It can be seen that grounding the inductor and the capacitor produces different results. In both cases, the resonance is suppressed, but to varying degrees. Greater suppression is achieved when the outer end of the inductor is grounded, and the difference becomes more pronounced as the grounding resistance decreases. When both capacitors are grounded, the coupling between the resonators is reduced, while the structure of the inductors remains largely unchanged. The inductors retain their original inductance, and the capacitance to ground is only slightly altered. As a result, the resonators still satisfy the conditions for resonance, albeit at a slightly shifted frequency. On the other hand, grounding the outer end of both inductors significantly alters their electrical parameters, and furthermore, by grounding the outer part of the inductors, the coupling with the adjacent resonator is significantly reduced.

Further simulations using the circuit model showed that grounding only one capacitor or only one inductor resulted in a slightly reduced suppression effect, as expected.

It should be noted that while the circuit model provides a useful starting point for further filter design, it has limitations in accurately capturing parasitic effects at higher frequencies. As shown in previous works on this topic, as the operating frequency increases, the physical dimensions of elements decrease to the point where they cannot be described as elements with concentrated parameters with sufficient accuracy. Some elements, e.g., inductors, begin to behave like transmission lines and must be described using distributed parameter models. Nevertheless, the results obtained from the circuit model validate the proposed method for resonator adjustment. For precise performance evaluation, full-wave 3D electromagnetic simulations using appropriate software tools are necessary.

## 4. Reconfigurable Dual-Band Bandpass Filter

**Figure 10:**
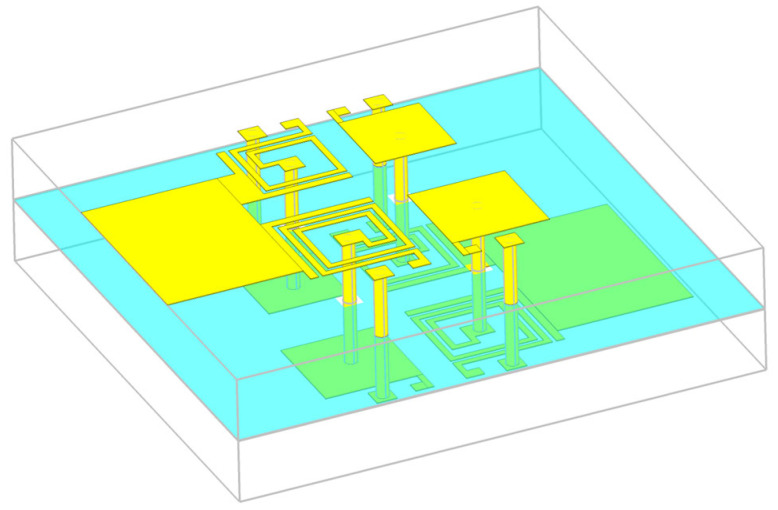
Reconfigurable dual-band bandpass filter using multilayer technology: perspective view with transparent substrate layers (ground layer is shown in blue color).

A reconfigurable dual-band bandpass filter can be designed by combining two bandpass filters operating in different bands, along with additional elements to enable reconfiguration. The selected center frequencies are 3.5 GHz and 5.8 GHz, corresponding to the 5G N78 band and Wi-Fi applications, respectively. This dual-band filter is designed using two independent filters arranged in a parallel configuration with common feeders. The layout of this configuration is shown in Figure 10.

The proximity of the two filters affects their performance due to mutual coupling. It has been observed that greater separation between the resonator pairs leads to increased attenuation in the stopbands. This effect becomes more pronounced at higher frequencies. To balance improved stopband suppression with overall circuit compactness, the spacing between the two filter sections was chosen as a compromise between electrical performance and footprint area.

To enable reconfigurability, additional elements must be incorporated into the non-reconfigurable filter design, including memristor pads, connections from the memristors to the inductor and capacitor, and extra vias to ground.

**Figure 11:**
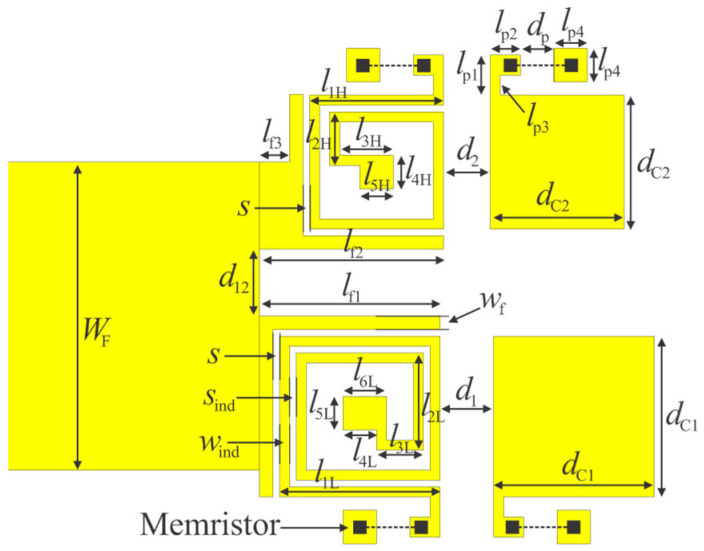
Reconfigurable dual-band bandpass filter with quasi-lumped elements: layout with dimensions.

Two pads were added near each inductor and patch capacitor, with dimensions of 300 µm × 300 µm and 500 µm × 500 µm, spaced 500 µm apart. These pads are intended for mounting memristors in standard 0603 surface-mount packages (0.6 mm × 0.3 mm) [2]. The larger pad serves as a connection point for a via that connects to the ground layer. The smaller pads are connected to either the inductors or the patch capacitors. The capacitances *C*_pad1_, *C*_pad2_, and *C*_g_, shown in Figure 7, represent the parasitic capacitances of the pads to the ground and the inter-pad capacitance, respectively. As expected, the addition of these elements slightly influenced the filter’s performance. Specifically, the added parasitics led to a small increase in both the capacitance of the patch capacitors and the inductance of the spiral inductors, resulting in a downward shift in the resonant frequencies. To compensate for these shifts, the inductors were shortened from the center. Minor adjustments to the spacing between resonators were also made to achieve the desired *S*_21_ and *S*_11_ characteristics. The final layout of the filter 3D model, incorporating all design elements, is shown in Figure 11. The geometric dimensions of the final filter layout are listed in Table 3. The proposed filter occupies a footprint of 0.10 λ_g_ × 0.12 λ_g_, where λ_g_ is the guided wavelength of a 50 Ω microstrip line at the center frequency of the lower passband, which is about 6.2 mm × 7.4 mm (not including the feed lines).

Our approach involves placing a memristor as a switching element on each inductor and capacitor, resulting in a total of eight memristors. Each memristor can be independently set to either the ON or OFF state. In the ON state, the memristor is emulated with a resistor (*R*_mem_ = 3 Ω), while in the OFF state, it is emulated with a capacitor (*C*_mem_ = 1.37 fF). Since each memristor can be individually controlled (i.e., to be in the ON or OFF state) there are 256 possible ON/OFF configurations. However, by considering the symmetry of certain combinations, and excluding some combinations based on logical conclusions, this number can be reduced. For example, to suppress a specific passband, it is sufficient to ground the resonator elements associated with that band. This can be achieved by any of the following options: (1) grounding both inductors, (2) grounding both capacitors, or (3) grounding the inductor of one resonator and the capacitor of the other resonator. This way, we reduced the combinatorial space to 22 cases, as shown in Table 4. In the table, the letter “G” indicates that the corresponding element is grounded by setting the associated memristor to the ON state.

The abbreviations used have the following meaning: the first letter, *C* or *L*, denotes the type of element on which the memristor is placed—a capacitor or inductor. The superscript 1 or 2 denotes the plane (upper or lower) where the memristor is placed, although the structure is symmetrical with respect to the ground layer. The subscript LB or HB refers to the resonator associated with the memristor, either for the low-frequency passband or the high-frequency passband.

There are four operating modes: dual-bandpass (DB), low-bandpass (LB), high-bandpass (HB), and bandstop (BS).

**Figure 12:**
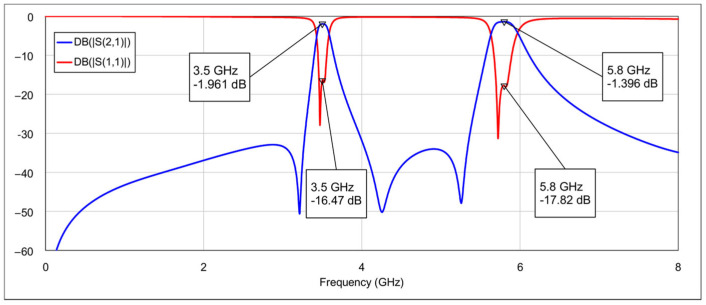
Simulated frequency response of the reconfigurable filter in dual-bandpass mode.

In the first operating mode, the 3D EM simulation results with all memristors in the OFF state are shown in Figure 12. This configuration corresponds to combination 1, depicted in Table 4. The center frequencies are 3.5 GHz and 5.8 GHz for the low and the high band, respectively. The 3 dB fractional bandwidths are 4.6 % and 5.8 % for the low passband and the high passband, respectively, while the insertion losses at center frequencies are 2 dB (3.5 GHz) and 1.4 dB (5.8 GHz). The return losses exceeded 16.4 dB and 17.8 dB at 3.5 GHz and 5.8 GHz, respectively. The out-of-band suppression level is greater than 30 dB.

**Figure 13:**
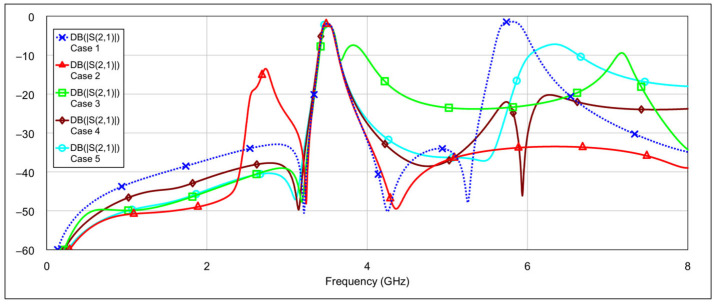
Simulated frequency responses of the reconfigurable filter in low-bandpass mode for various combinations of high-frequency band memristor states (cases 2–5).

**Figure 14:**
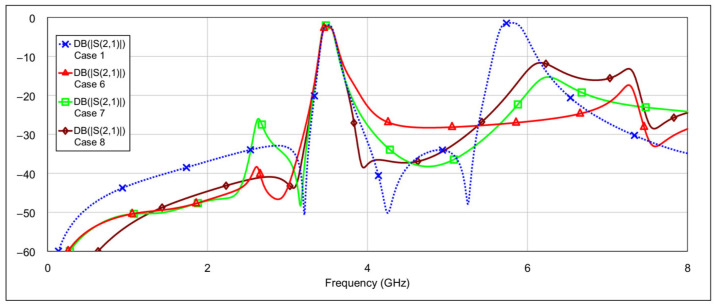
Simulated frequency responses of the reconfigurable filter in low-bandpass mode for various combinations of high-frequency band memristor states (cases 6–8).

**Figure 15:**
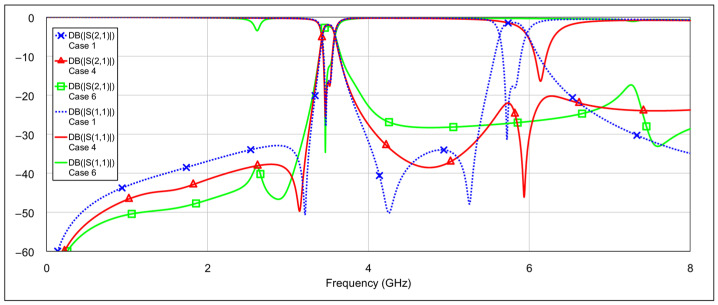
The best simulated results for the low-bandpass mode observed among all tested configurations of high-frequency band memristor states (cases 4 and 6).

The second operating mode is the low-bandpass mode, achieved by disabling the high-frequency resonators, specifically by grounding the inductor and/or capacitor of the high-frequency band resonators. Figure 13 and Figure 14 show the *S*-parameters for combinations 2–5 and 6–8 from Table 4, respectively. These configurations yield varying results. Among them, combinations 4 (grounding the inductor of one resonator and the capacitor of the other) and 6 (grounding both the inductor and capacitor of a single resonator) produce the most acceptable filter characteristics. In contrast, fully grounding both high-frequency resonators results in degraded performance. Importantly, these combinations do not significantly affect the passband characteristics at 3.5 GHz. The results of the two best-performing configurations are shown in Figure 15.

**Figure 16:**
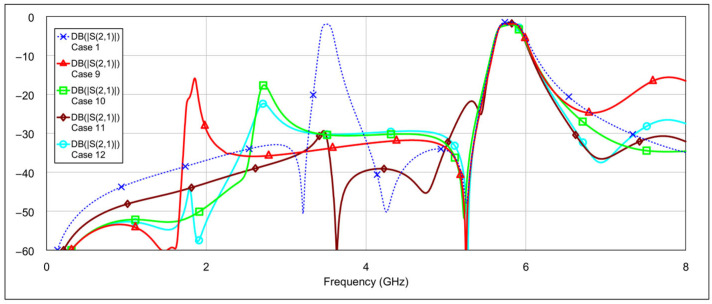
Simulated frequency responses of the reconfigurable filter in high-bandpass mode for various combinations of low-frequency band memristor states (cases 9–12).

**Figure 17:**
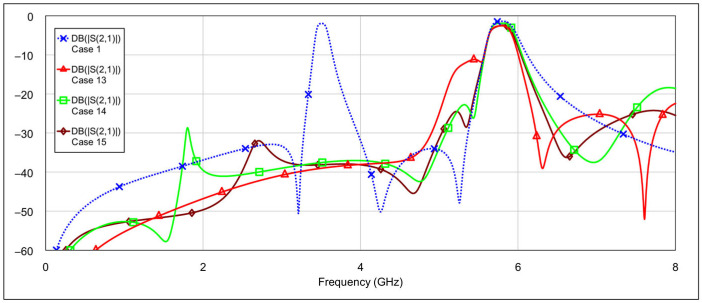
Simulated frequency responses of the reconfigurable filter in high-bandpass mode for various combinations of low-frequency band memristor states (cases 13–15).

**Figure 18:**
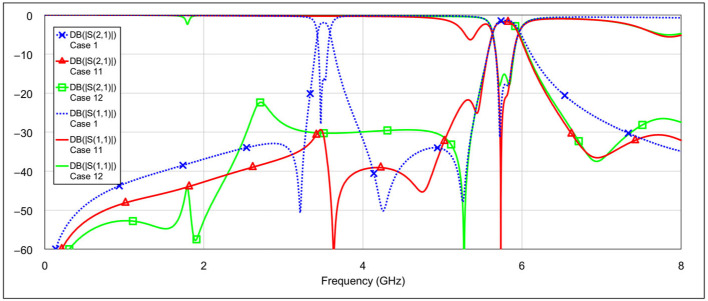
The best simulated results for the high-bandpass mode, for two combinations of low-frequency band memristor states (cases 11 and 12).

The third operating mode is high-bandpass mode, achieved by grounding the inductor and/or capacitor of the low-frequency band resonators, using a similar approach as in the previous mode. Figure 16 and Figure 17 show the *S*-parameters for combinations 9–12 and 13–15, respectively, as shown in Table 4. As expected, different configurations yield varying results. Acceptable performance is observed for combinations 11 (grounding the inductor of one resonator and the capacitor of the other) and 12 (grounding both the inductor and the capacitor of a single resonator). Interestingly, fully grounding both low-frequency resonators provides the best rejection in the low-frequency band, but it negatively impacts the high-frequency band performance by eliminating the left-hand transmission zero. Similarly to the second operating mode, none of the tested configurations significantly degrade the 5.8 GHz bandpass characteristics. The results of the two best-performing configurations are shown in Figure 18.

**Figure 19:**
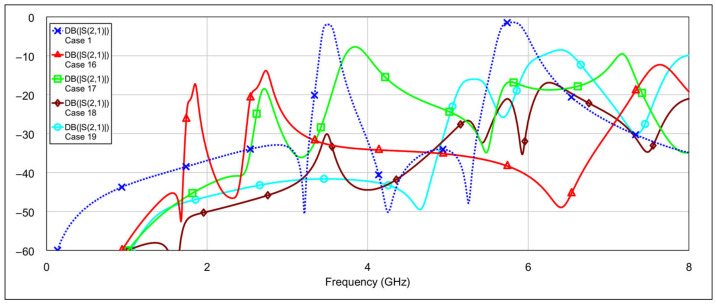
Simulated frequency responses of the reconfigurable filter in bandstop mode for various combinations of low-frequency and high-frequency band memristor states (cases 16–19).

**Figure 20:**
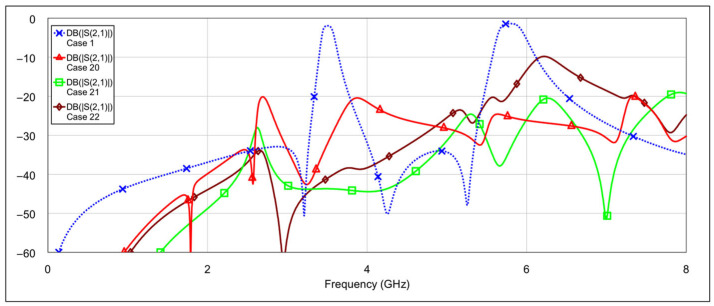
Simulated frequency responses of the reconfigurable filter in bandstop mode for various combinations of low-frequency and high-frequency band memristor states (cases 20–22).

**Figure 21:**
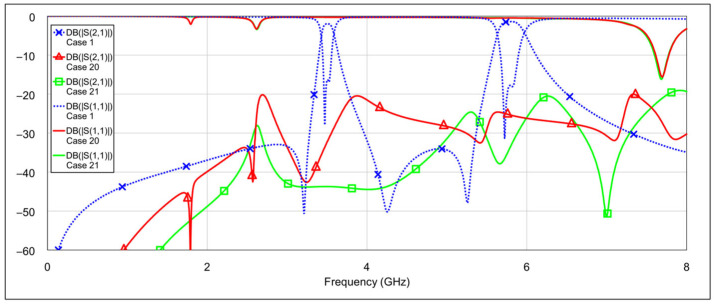
The best simulated results for the bandstop mode (cases 20 and 21).

The fourth operating mode is the bandstop (BS) mode. Figure 19 and Figure 20 show the *S*-parameters for combinations 16–19 and 20–22, respectively, from Table 4. The best results are achieved using combination 20 (grounding both the inductor and capacitor of a single resonator for both low-frequency and high-frequency bands) and combination 21 (where all elements are grounded except for one capacitor on the low-frequency band resonator and one capacitor on the high-frequency band resonator). These two cases are shown separately in Figure 21. As seen in the results, the attenuation across the entire 0–8 GHz frequency range is at least 19 dB, with attenuation exceeding 25 dB in the 3-dB passbands of both the low- and high-frequency bands.

## 5. Comparison of Filter Characteristics for Using PIN Diode Instead of Memristor as Switching Element

**Figure 22:**
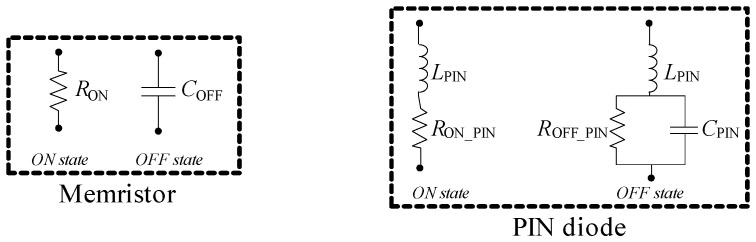
Equivalent circuit models of the memristor and the PIN diode.

Due to the existence of commercially available PIN diodes, in contrast to memristors, various structures proposed in the literature use PIN diodes as switching elements. These studies demonstrate the feasibility of controlling filter passbands through PIN diode-based switching. In our work, we also aimed to investigate whether PIN diodes could be used in place of memristors for realizing a reconfigurable bandpass filter. For this purpose, we performed a 3D EM analysis of all four operating modes of the presented filter, substituting the memristor equivalent circuit model with that of a PIN diode. The biasing circuit for the PIN diode is beyond the scope of this analysis. For the diode model, we used the electrical parameters provided in the manufacturer’s datasheet, specifically for the MA4PBL027 device. Figure 22 shows the equivalent circuit models of both the memristor and the PIN diode, for both ON (conducting) and OFF (non-conducting) states. The parameters of the memristor are *R*_ON_ = 3 Ω and *C*_OFF_ = 1.37 fF, while the PIN diode parameters are *L*_PIN_ = 0.15 nH, *R*_ON_PIN_ = 0.05 Ω, *R*_OFF_PIN_ = 100 kΩ, and *C*_PIN_ = 40 fF. The physical dimensions of the MA4PBL027 diode are compatible with the space allocated for SMD components in our 3D model. In the ON state, the PIN diode can be modeled as a series connection of an inductance and low resistance, whereas in the OFF state, it can be modeled as a high resistance in parallel with a capacitance. As will be shown, this OFF-state capacitance has the most significant impact on the filter’s performance.

**Figure 23:**
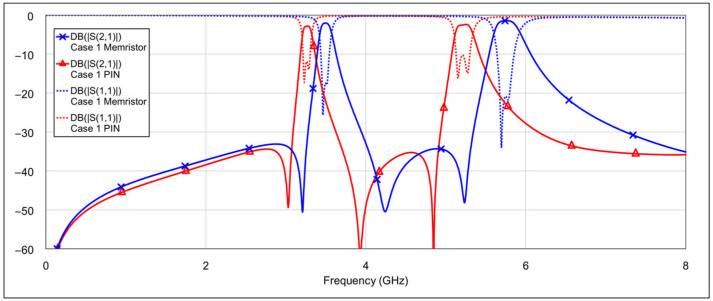
Comparison results: Memristor vs. PIN diode performance in the dual-bandpass mode (case 1).

**Figure 24:**
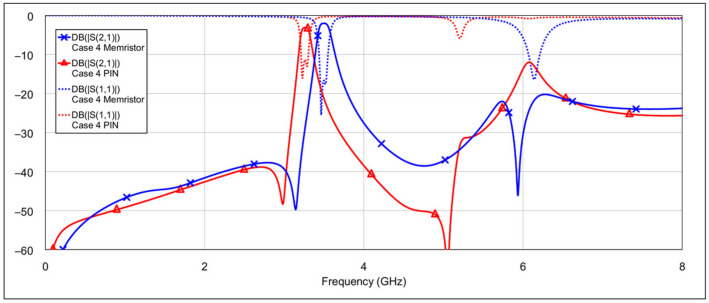
Comparison results: Memristor vs. PIN diode performance in low-bandpass mode (case 4).

**Figure 25:**
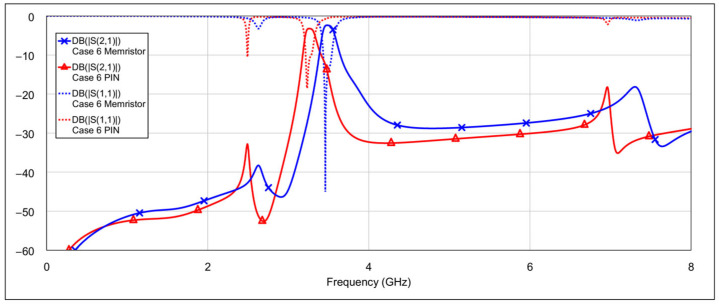
Comparison results: Memristor vs. PIN diode performance in the low-bandpass mode (case 6).

**Figure 26:**
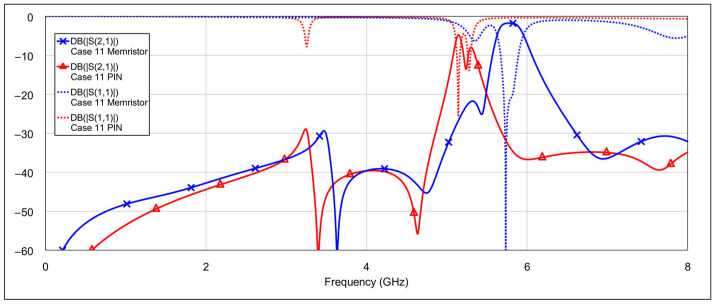
Comparison results: Memristor vs. PIN diode performance in the high-bandpass mode (case 11).

**Figure 27:**
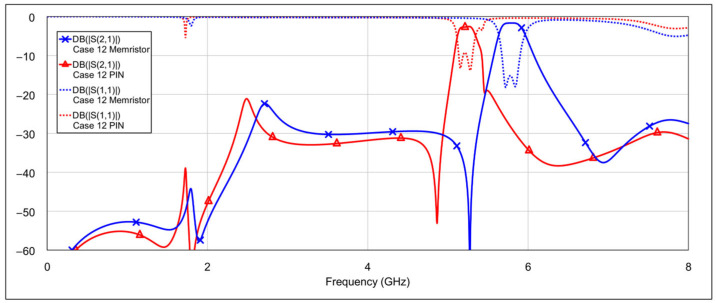
Comparison results: Memristor vs. PIN diode performance in the high-bandpass mode (case 12).

**Figure 28:**
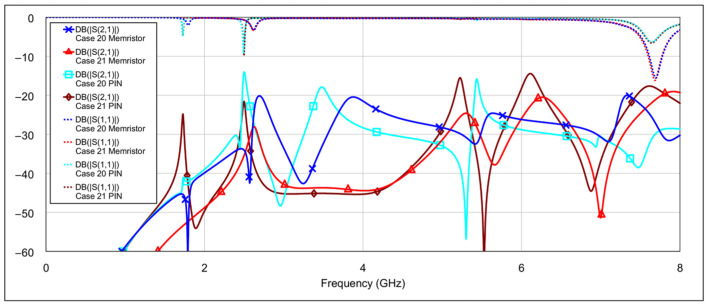
Comparison results: Memristor vs. PIN diode performance in the bandstop mode (cases 20 and 21).

An analysis was performed for all four filter modes, and the results are shown in Figure 23, Figure 24, Figure 25, Figure 26, Figure 27 and Figure 28. The most noticeable effect observable in the dual-bandpass, low-bandpass, and high-bandpass modes is a shift of the passbands toward lower frequencies. This phenomenon is caused by the relatively large OFF-state capacitance of the PIN diode. Specifically, the OFF-state capacitance of the PIN diode (40 fF) is approximately 30 times greater than that of the memristor (1.37 fF). This capacitance is on the same order of magnitude as the spiral inductor’s capacitance (*C_L_*_1_, *C_L_*_2_) and is also comparable to the patch capacitor capacitance (*C_C_*). The resulting increase in total capacitance leads to a decrease in the center frequency of the filter. It may be possible to compensate for this frequency shift by reducing the length of the spiral inductor (thus lowering its inductance) or by decreasing the dimensions of the patch capacitor. However, such geometric modifications could introduce unwanted side effects. A detailed investigation of these trade-offs should be considered in future research.

In addition to the frequency shift, a slightly higher insertion loss within the passband is also observed. This can again be attributed to the larger OFF-state capacitance of the PIN diode, which has an influence on coupling between resonators due to its placement at the end of the spiral inductor. In the high-bandpass mode, particularly in case 11, the passband characteristics are significantly degraded. Out-of-band characteristics are generally slightly worse when using PIN diodes, compared to memristors. This is most pronounced in the bandstop mode.

Taking all of the above into consideration, it can be concluded that the use of the PIN diode in our case is not an optimal solution, although the diode used in the analysis belongs to a class of devices with relatively low capacitance compared to other PIN diodes. The compact dimensions of the proposed filter make it highly sensitive to additional parasitic capacitances introduced by external components. In larger structures, the capacitance of a PIN diode may not pose a significant issue; however, in our case, it has a substantial impact on filter performance. In our case, the use of memristor shows a great advantage over PIN diode as a switching element.

We conducted a comparison of our filter implementation with the most recent relevant publications, as illustrated in Table 5. This comparison encompasses several design and performance parameters, including filter mode, central frequencies, 3 dB fractional bandwidths, insertion losses and return losses at the central frequencies, number of switches, and circuit dimensions (i.e., footprint area). The proposed filter features a compact size with the smallest footprint area, compared to all other filters. Additionally, the advantage of our design is four operating modes, an attribute shared by only two other narrow-band filter implementations.

## 6. Conclusions

This paper presents a novel design of a reconfigurable dual-band bandpass filter implemented using multilayer technology and memristors as switching elements.

Memristors are very valuable components due to their superior characteristics compared to the other commonly used switching components, such as PIN diodes, FET, MEMS, etc. Memristors are non-volatile, feature nanoscale dimensions, exhibit low insertion loss in conducting state, provide high isolation in non-conducting state, consume low power and do not require a continuous bias voltage. Given those advantages, memristors are excellent candidates for use in modern wireless microwave circuits.

Filters based on multilayer technology with quasi-lumped elements were chosen as the basis for this design. Their most important advantage is the significant miniaturization of the overall filter footprint. To enable reconfigurability, additional components were incorporated into the initial filter structure, including pads for mounting memristors and vias for connecting them to the ground layer. In the simulations, the memristor was modeled as a two-state element: the ON state was represented by a resistance of 3 Ω and the OFF state by a capacitance of 1.37 fF.

For the filter design, we selected a dual-band bandpass filter operating at 3.5 GHz and 5.8 GHz. The dual-band filter was realized as two independent filters arranged in a parallel configuration with common feeders. The design concepts were initially evaluated using an equivalent circuit model, where the impact of memristor placement on filter performance was examined. Following this, a full 3D model of the dual-band filter was developed, and detailed analyses were conducted through 3D EM simulations.

The filter is designed for center frequencies of 3.5 GHz and 5.8 GHz, corresponding to the low-bandpass and high-bandpass, respectively. The realized filter exhibits the following performance characteristics: 3 dB fractional bandwidths of 4.6% for the low passband and 5.8% for the high passband, insertion losses of 2 dB and 1.4 dB at the respective center frequencies, and return losses exceeding 16.4 dB and 17.8 dB at 3.5 GHz and 5.8 GHz, respectively. The filter occupies a footprint area equal to 0.10 λ_g_ × 0.12 λ_g_, where λ_g_ is the guided wavelength of a 50 Ω microstrip line at the center frequency of the lower passband (approximately 6.2 mm × 7.4 mm, excluding the feed lines).

The filter supports four operating modes: dual-bandpass (DB), low-bandpass (LB), high-bandpass (HB), and bandstop (BS). The frequency responses of the filter for various memristor state combinations were presented in detail, and the most effective configurations for each of the four operating modes were identified.

Each passband can be controlled independently, with minimal impact on the frequency response of the other band.

It has also been demonstrated that, in the case of miniature structures such as the one presented here, memristors offer a clear advantage over PIN diodes as switching elements.

## Figures and Tables

**Table 1:** Geometric parameters of the second-order bandpass filter. Dimensions are in mm.

***W*** **_F_**	***w*** **_f_**	***l*** **_f_**	***s***	***s*** **_ind_**	***l*** **_1_**
4.6	0.2	2.7	0.1	0.1	2.4
***l*** **_2_**	***l*** **_3_**	***l*** **_4_**	***l*** **_5_**	***d*** **_12_**	***d*** **_C_**
0.9	1.88	0.5	0.5	0.4	2.4

**Table 2:** Values of the elements used in the circuit model from Figure 5 (capacitances in pF, inductance in nH, resistance in Ω).

Band	*L_L_*	*R*_L_	*C_L_*_1_, *C_L_*_2_	*C*_IC_	*C_C_*	*C_LC_*	*C*_F_
3.5 GHz	17.5	1	0.015	0.1	0.2	0.01	0.13

**Table 3:** Geometric parameters of the dual-band bandpass filter. Dimensions are in mm.

***W*_F_**	***w*_f_**	***l*_f1_**	***l*_f2_**	***l*_f3_**	***d*_12_**	***s***	***s*_ind_**	***w*_ind_**	***l*_1L_**
4.6	0.2	2.7	2.75	0.45	1.0	0.1	0.1	0.15	2.40
***l*_2L_**	***l*_3L_**	***l*_4L_**	***l*_5L_**	***l*_6L_**	***l*_1H_**	***l*_2H_**	***l*_3H_**	***l*_4H_**	***l*_5H_**
1.45	0.7	0.7	0.5	0.65	2.0	0.8	0.8	0.5	0.5
***d*_1_**	***d*_2_**	***d*_C1_**	***d*_C2_**	***l*_p1_**	***l*_p2_**	***l*_p3_**	***l*_p4_**	***d*_p_**	
0.8	0.7	2.4	2.0	0.6	0.45	0.3	0.5	0.5	

**Table 4:** Filter modes depending on the memristors’ states.

No.	*C* ^1^_HB_	*L* ^1^_HB_	*C* ^2^_HB_	*L* ^2^_HB_	*C* ^1^_LB_	*L* ^1^_LB_	*C* ^2^_LB_	*L* ^2^_LB_	Mode
1	-	-	-	-	-	-	-	-	DB
2	-	G	-	G	-	-	-	-	LB
3	G	-	G	-	-	-	-	-	LB
4	G	-	-	G	-	-	-	-	LB
5	G	G	G	G	-	-	-	-	LB
6	-	-	G	G	-	-	-	-	LB
7	-	G	G	G	-	-	-	-	LB
8	G	-	G	G	-	-	-	-	LB
9	-	-	-	-	-	G	-	G	HB
10	-	-	-	-	G	-	G	-	HB
11	-	-	-	-	G	-	-	G	HB
12	-	-	-	-	-	-	G	G	HB
13	-	-	-	-	G	G	G	G	HB
14	-	-	-	-	-	G	G	G	HB
15	-	-	-	-	G	-	G	G	HB
16	-	G	-	G	-	G	-	G	BS
17	G	-	G	-	G	-	G	-	BS
18	G	-	-	G	G	-	-	G	BS
19	G	G	G	G	G	G	G	G	BS
20	-	-	G	G	-	-	G	G	BS
21	-	G	G	G	-	G	G	G	BS
22	G	-	G	G	G	-	G	G	BS

**Table 5:** Comparison with recent publications of reconfigurable filters.

Refs.	No. Modes	Modes	Centr. Freq. (GHz)	FBW (3 dB)	Insertion Loss (dB)	Return Loss (dB)	No. Switches	Footprint Area λ_g_ × λ_g_
[20]	4	2-BPF LowBPF HighBPF BSF	2.4 and 5.2 2.4 5.2 /	0.10 and 0.06 0.103 0.06 /	2.06 and 3.5 2.4 3.43 35 (˂8.5 GHz)	12 and 12 13 13 /	PIN: 4	0.43 × 0.36 Exp.
[21]	3	Wide-BPF 2-BPF 3-BPF	1.5 0.38 and 2.68 0.37 and 1.54 and 2.68	0.37 0.21 and 0.16 0.20 and 0.27 and 0.18	<0.5 <0.4 <0.6	>26 >20 >18	PIN: 7	0.75 × 0.4 Exp.
[22]	3	Wide-BPF 2-BPF BSF	2 1.5 and 2.5 2	0.85 0.4 and 0.25 0.3	0.9 1.1 and 1.5 32	>21.5 >20 >20	PIN: 6	0.61 × 0.27 Exp.
[15]	4	2-BPF LowBPF HighBPF BSF	2.15 and 3.1 2.15 3.1 /	0.09 and 0.09 0.102 0.095 /	1.25 and 1.3 1.25 1.3 >19 (˂4.5 GHz)	>15 >20 >20 /	Mem: 2/ PIN: 2	0.63 × 0.43 Exp. PIN
[23]	3	Wide-BPF BSF 2-BPF	1.92 1.92 1.58 and 2.23	0.53 0.3 (FBW-10 dB) 0.04 and 0.03	0.9 15.5 2 and 2.2	>12 / >12	PIN: 6	0.44 × 0.29 Exp.
[14]	2	2-BPF LowBPF	2.42 and 5.2 2.42	0.08 and 0.054 0.076	2.3 and 3.1 2.2	>15 >15	Mem: 2	0.37 × 0.18 Exp. emulated
This work	4	2-BPF LowBPF HighBPF BSF	3.5 and 5.8 3.5 5.8 /	0.046 and 0.06 0.046 0.06 /	2.0 and 1.4 2.0 1.4 20 (˂8.0 GHz)	16.4 and 17.8 16.4 17.8 /	Mem: 8	0.10 × 0.12 Sim.

## Data Availability

Data are contained within the article. The data presented in this study are available in this article.
